# English language proficiency and the accommodations for language non-concordance amongst patients utilizing chiropractic college teaching clinics

**DOI:** 10.1186/2045-709X-21-7

**Published:** 2013-02-01

**Authors:** Richard P Saporito

**Affiliations:** 1 Assistant Professor of Clinical Sciences, University of Bridgeport College of Chiropractic, 75 Linden Avenue, Bridgeport, CT 06604, USA

**Keywords:** English language proficiency, Language concordance, Accommodations, Informed consent, Chiropractic teaching clinics

## Abstract

**Background:**

The number of households in the United States that are not proficient in the English language is growing and presenting a challenge to the health care system. Over nineteen percent of the US population speak a language other than English in the home. This increase in language discordance generates a greater need to find and implement accommodations in the clinical setting to insure accurate and efficient diagnosis and treatment as well as provide for patient safety. Aim: The purpose of this study is to determine the percentage of patients accessing the chiropractic college teaching clinics who are not proficient in the English language and to what extent the colleges provide accommodations for that language disparity.

**Methods:**

The clinic directors and deans of the Association of Chiropractic Colleges were surveyed via an on-line survey engine. The survey queried the percentage of the patient population that is not English language proficient, the accommodations the college currently has in place, if the college has a language specific consent to treat document and if the college has a written policy concerning patients without English proficiency.

**Results:**

Fifty percent of the contacted chiropractic colleges responded to the survey. In the respondent college clinics 16.5% of the patient population is not proficient in English, with over 75% speaking Spanish. All but one of the respondents provide some level of accommodation for the language non-concordance. Forty five percent of the responding colleges employ a language specific consent to treat form. The implementation of accommodations and the use of a language specific consent to treat form is more prevalent at colleges with a higher percentage of non-English speaking patients.

**Conclusions:**

The percentage of patients with limited English proficiency accessing services at the teaching clinics of the chiropractic colleges mirrors the numbers in the general population. There is a wide disparity in the accommodations that the individual colleges make to address this language discordance. There is a need to further develop accurate and meaningful accommodations to address language disparity in the chiropractic teaching clinics.

## 

The ethnic diversity in the United States is expanding. Persons born outside the country comprise 12.5% of the resident population with just under one third arriving in the past 10 years [1]. In addition to those whom have entered the United States legally, there is an estimated 10.8 million additional persons who have equivocal resident status. Coincident with this change in ethnic composition is an increase in the number of households in which the primary language is not English. The 2010 U.S. Census reveals that over 55 million individuals, 19.9% of all those 5 years of age and older, speak a language other than English in the home with 45% indicating that they speak English less than “Very Well” [1].

This deficiency in English language proficiency may have a significant impact on the health care system. Quality patient care is dependent upon obtaining a thorough history, accurate feedback during the physical examination and a valid informed consent. Without a reasonable level of communication with the patient, compliance with treatment recommendations may also become problematic [2,3]. Effective communication has a positive effect on symptom response, pain control and physiological and functional improvement [4-7].

The risk of adverse effects of treatment is increased by communication difficulties. Divi, et al. found that 49.1% of adverse events involving patients with limited English language proficiency resulted in some level of physical harm, versus 29.5% in English speaking patients. Further, in those patients experiencing detectable physical harm, the level of that harm was more significant in the English non-proficient patients with 46.8% suffering, “moderate temporary harm,” or worse, compared to 24.4% in those speaking English [8].

When services are provided to address language disparities, the nature of the interpreter service can affect the overall clinical outcome. Flores, et al. found that in all encounters involving language interpreters, errors of translation occurred. They found no statistical difference in the frequency of error attributable to whether the translator was a professional or ad hoc. Their analysis of the error rates revealed that 52% were errors of omission, 16% false fluency, 13% substitution, 10% editorializing and 8% additions. Sixty-three percent of the translational errors were potentially of clinical significance with the incidence 1.45 times more likely when the translation was performed by an ad hoc interpreter versus a professional translator [9]. Jackson, et al., found a similar distribution of error type although the clinical significance was much lower. They attribute this to a “best case” scenario where the interpreter was professionally trained, the topic was familiar and the doctor / patient relationship was established [10].

The level of satisfaction with care in patients without English language proficiency is significantly associated with the level of language concordance [11,12]. Ferguson and Candib, in a review of the literature, present evidence that when the physician and patient speak the same language the patient receives more information concerning his or her health and treatment and is more likely to be encouraged to participate in medical decision making [11]. Patients report an expectation of information concerning their condition and express dissatisfaction when they perceive that the lack of information from their physician has limited their participation in their care [11,13,14]. Carlesso, et al., in assessing the patient’s perspective on adverse events involving manual therapy, found that contextual factors such as education concerning the nature and possibility of adverse events and trust in the provider improved the patient’s attitude about the adverse event [6]. The trust patients express towards their physician is directly dependent on the degree to which they perceive socio-ethnic concordance with the physician [15,16]. Patient satisfaction has also been shown to be associated with the method used to address language issues. Lee, et al. found the greatest encounter satisfaction in Spanish speaking patients with a professional translator via a telephone service and with language concordant providers, and the least satisfaction when translation was provided by a family member or ad hoc interpreter [17]. The chiropractic profession places a high regard on the issue of satisfaction and often references studies demonstrating the high level reported by patients [18-20]. Incorporating accommodations to cultural and language dis-concordance in the teaching clinics would be consistent with this focus.

Currently no literature exists on the effect of language disparity in the chiropractic college teaching clinics. Many of the chiropractic colleges in the United States have outpatient care facilities located in ethnically diverse population centers and, as such, may serve a population with a variable level of English language proficiency. The purpose of this study is to establish baseline data on the utilization of chiropractic teaching clinics by non-English language proficient patients and the resources those clinics provide to address language non-concordance.

## Methods

**Figure 1:**
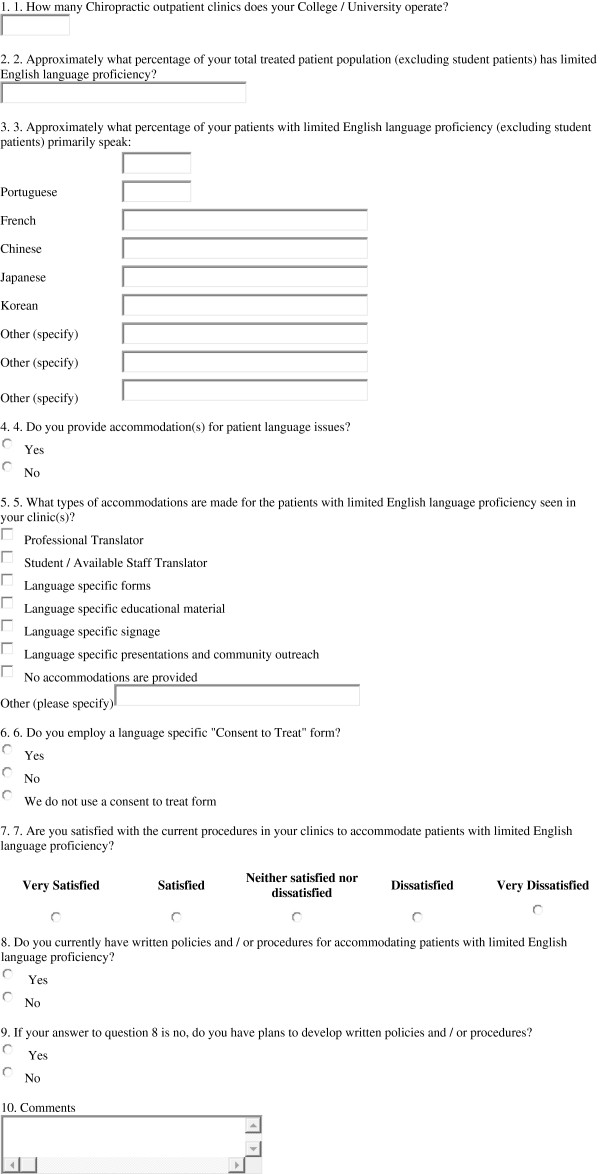
The survey instrument.

A survey instrument consisting of 9 questions was posted on an on-line survey engine (http://www.SurveyMonkey.com), allowing for anonymity of the respondents. The study design and survey instrument were approved for use by the Institutional Review Board of the University of Bridgeport. A request to complete the survey was sent to the clinic directors and / or deans of the member colleges of the Association of Chiropractic Colleges. The request asked the person filling out the survey to aggregate data from all clinics that the college directly administrates. Community based clinical training sites, hospital rotations and all other clinical training opportunities not under the direct control of the chiropractic college were excluded. While these alternative training sites may provide a greater exposure to language diversity, the college may not be able to affect accommodations for those disparities. The survey consisted of open ended, multiple choice, categorical and Likert-scale questions (Figure 1). A 5 level Likert scale was used to assess the level of satisfaction each respondent had with his or her institution’s language accommodation strategies. The responses were quantified by assigning a value of −2 for Very Dissatisfied, -1 for Dissatisfied, 0 for Neither Satisfied or Dissatisfied, +1 for Satisfied and +2 for Very Satisfied. Approximately one month following an initial request for participation a second request was sent via electronic mail.

Respondents were given the following choices of accommodations for patients with limited English language proficiency and asked to indicate which are used in their clinic(s): professional translator, student or staff (ad hoc) translator, language specific forms, language specific educational materials, appropriate signage and educational presentations.

## Results

Nine responses were obtained from the 18 colleges contacted for a response rate of 50%. The nine colleges participating represent a total of 37 clinical locations ranging from one to eight with a mean of five. The percentage of patients with limited proficiency in English varied widely from a low of 1% at one institution to a high of 50% at another. One college responded with a range of 10 – 15%. For statistical purposes this was averaged to 12.5%. The average percent of the limited English language population is 16.5% across all respondent institutions. There appears to be no correlation between the number of clinic sites and the concentration of language non-concordance. The college reporting the highest percentage of non-English language proficient patients only operates one clinical site while the school with the greatest number of clinical locations, eight, indicates a non-English proficient population of 5%. The Spanish language comprises the greatest cohort of non-English language proficient patients making up 75.6% of the total. Asian languages made up the majority of the remainder constituting 19.5% of the population. The remaining 4.9% were not specifically identified by the respondents (Table 1).

**Table 1:** Survey results

**Question**		**School**	**1**	**2**	**3**	**4**	**5**	**6**	**7**	**8**	**9**
**1**	**# of clinics**		**1**	**4**	**1**	**8**	**1**	**6**	**5**	**6**	**5**
**2**	**% non-English**		**50**	**1**	**10-15**	**5**	**5**	**20**	**25**	**10**	**20**
**3**	**Language**	**Spanish**	**80**	**90**	**0**	**100**	**85**	**40**	**100**	**95**	**90**
		**Portuguese**	**0**	**0**	**0**	**0**	**0**	**0**	**0**	**0**	**0**
		**French**	**1**	**0**	**1**	**0**	**0**	**0**	**0**	**0**	**0**
		**Chinese**	**5**	**0**	**40**	**0**	**0**	**15**	**0**	**0**	**0**
		**Japanese**	**1**	**0**	**1**	**0**	**0**	**10**	**0**	**0**	**0**
		**Korean**	**2**	**0**	**55**	**0**	**15**	**5**	**0**	**0**	**10**
		**Other**	**1**	**10**	**3**	**0**	**0**	**30**	**0**	5*	**0**
**4**	**+ Accommodations**		**N**	**N**	**Y**	**Y**	**nr**	**Y**	**Y**	**Y**	**Y**
**5**	**Method**	**Professional**	**N**	**N**	**N**	**N**	**N**	**N**	**Y**	**N**	**N**
		**Student/Staff**	**N**	**N**	**Y**	**Y**	**Y**	**Y**	**Y**	**Y**	**Y**
		**Forms**	**N**	**N**	**N**	**Y**	**N**	**Y**	**Y**	**Y**	**Y**
		**Ed Material**	**N**	**N**	**N**	**Y**	**N**	**N**	**Y**	**N**	**Y**
		**Signs**	**N**	**N**	**N**	**N**	**N**	**N**	**Y**	**N**	**N**
		**Presentations**	**N**	**N**	**N**	**N**	**N**	**Y**	**N**	**N**	**√**
**6**	**Consent Form**		N	N	N	N	N	**Y**	**Y**	**Y**	**Y**
**7**	**Satisfaction**		**0**	**−1**	**0**	**1**	**0**	**1**	**1**	**1**	**1**
**8**	**Policy**		**N**	**N**	**N**	**N**	**N**	**Y**	**N**	**Y**	**Y**
**9**	**Plan**		**Y**	**N**	**Y**	**N**	**Y**	**n/a**	**N**	**n/a**	**n/a**
										*Vietnamese	

Y = a positive response.

N = a negative response.

n/r = no response provided.

**Figure 2:**
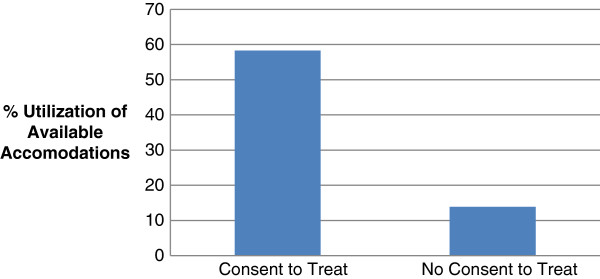
Accommodation utilization as a function of the use of a language specific consent to treat form.

Of the colleges reporting, 55.6% indicate that they do not use a language specific consent to treat form. An analysis of the colleges that do not employ a language specific form reveals that they utilize 13.9% of the available accommodations listed on the survey. The four colleges that do incorporate a language specific consent to treat form on average apply 58.3% of the available accommodations (Figure 2). Further, three of the four schools that have language specific consent to treat forms also have a written policy pertaining to addressing language discordance in their teaching clinics as compared to none of the five colleges without a language specific form. Of those five colleges, three report that they have a plan to develop a written policy.

On the question of the level of satisfaction with their institution’s accommodation strategies for patients that are non-English language proficient, five schools reported that they are, “Satisfied,” three reported, “Neither Satisfied nor Dissatisfied,” and one college responded they are, “Dissatisfied.” The average level of satisfaction for those colleges who have implemented a language specific consent to treat form is, “Satisfied,” (a Likert score of +1) while an average response of, “Neither Satisfied nor Dissatisfied,” (a Likert scores of zero) is obtained for the five schools not employing a language specific form.

**Figure 3:**
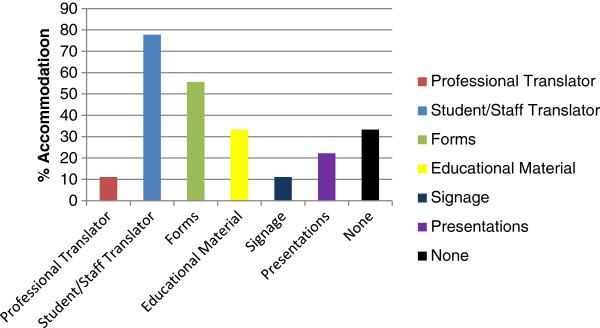
Language accommodation stratagies.

There also exists a wide disparity in the methods by which the different institutions accommodate the language needs of their patient populations. Figure 3 illustrates the responses for this survey question. The most prevalent strategy, employed by 77.8% of the colleges, is to utilize existing staff, including interns, with multilingual ability, as translators. Five of the nine respondents have language specific forms with 33.3% also incorporating translated patient educational materials. Two of the institutions provide patient education presentations for non-English proficient patients. Two of the colleges responding, 22.2%, report that they do not provide any accommodations for their non-English speaking patient population. Only one institution reported employing a professional translator.

## Discussion

Language concordance is an important factor in patient compliance, treatment efficacy and risk management. A failure to adequately address a communication disparity can have significant impact on outcome and the frequency and severity of any adverse effects of treatment. Engaging the patient in an active discourse and insuring patient comprehension is an ethical imperative. The doctrine of informed consent states that the physician has an obligation, both ethical and legal, to have an appropriate conversation, or its equivalent, with the patient. According to the published policy statement by the general counsel of the American Medical Association, “It is a process of communication between a patient and physician that results in the patient's authorization or agreement to undergo a specific medical intervention.” The content of that conversation should be thorough enough, “…so that he or she can make an informed decision to proceed or to refuse a particular course of medical intervention.” [21].

Satisfaction is both a reflection of the patient’s perception of the efficacy of the specific interventions provided as well as their assessment of the interpersonal components of the doctor-patient encounter. To a limited extent the level of satisfaction a patient has can also influence outcome measurement. In their 2005 observational study on satisfaction as a predictor of outcome in patients enrolled in the UCLA low back pain study, Hurwitz, et. al. found a short term decrease in reported pain and a longer term improvement in disability in patients generally satisfied with the care they received. The study also found a greater level of satisfaction in patients receiving care from a chiropractic physician versus from a medical provider. This increased satisfaction was attributed to the greater amount of information about their condition and treatment that was provided by the chiropractor. The difference in satisfaction between provider types disappeared when the patient received more self-care advice and an explanation of their treatment [22].

The interpretation of the results of this survey are limited by the response rate. Given the small sample size, extrapolating the results to the entire profession should be done with caution. For the data acquired, there are some identifiable trends. The patient population served by the chiropractic clinics is slightly less representative of the language diversity present in the population at large, 16.5% versus 19.9%. There is a wide percentage distribution of non-English proficient patients across the clinical sites responding, from near zero to half of all patients served. Spanish is the predominant language constituting 75% of all the non-English language proficient population.

Less than half of the colleges responding provide any significant accommodation to address language non-concordance. One institution provides a professional translator, five schools provide clinic forms in language appropriate format and only four colleges utilize a non-English consent to treat form. The four responding institutions that do provide a language appropriate consent to treat form also provide a significantly greater number of other accommodations. Seventy-five percent of these schools also have a written policy concerning accommodations for non-English proficient patients.

The data show that the colleges that place a priority on addressing language non-concordance employ a number of different strategies, from professional translators to patient education. In comparing colleges that provide many accommodations to those that supply few there appears to be only a slight difference in the percentage of non-English language proficient patients served, (18.7% versus 14.7%). The results, however, may be skewed. One low accommodation college is also the institution with the highest percentage of non-English speaking patients (50%). If this response is excluded from the data, the difference in patient population served is 18.7% versus 5.9%. The observations from the survey bear out the expectation that chiropractic college teaching clinics that serve a higher percentage of patients that are not proficient in the English language employ more strategies to accommodate for that language disparity.

The most commonly employed methods of addressing language discordance is to use ad hoc interpreters; these may be other interns, staff members or family members. This is certainly a convenient and cost effective approach, but does it produce the same outcome in terms of clinical results and patient satisfaction? Seven of the nine reporting schools (77.8%) use this ad hoc strategy. There are a number of difficulties that emanate from this solution. The language ability of the ad hoc translator may not be adequate. There may exist a lack of understanding of the information being requested or provided by the physician / intern and by the patient. There can be a tendency of family members, when acting as a translator, to interject their own observations, opinions, interpretations and questions and to change the questions, comments and advice of the physician [23]. A similar tendency to alter the translation was found by Elderkin-Thompson et al. in their investigation of the use of nurses as interpreters in a primary care setting [24]. A study by Hunt and de Voogd revealed that some physicians, in response to the increased time associated with the use of ad hoc or undertrained interpreters, abbreviate their consultations to compensate for the time loss. The study further demonstrated that even though the translations provided were incomplete and inaccurate, the patients perceived the encounter as successful and felt they understood their condition and treatment [25].

Regardless of whether an institution employs a professional translator or relies on an ad hoc solution, training the physicians, interns and staff is an important consideration [9]. Hudelson, et al. found that 58.6% of the physicians in their study considered themselves, “highly competent,” at working with interpreters. When asked to identify good practice behaviors, those same, self-assessed, “highly competent,” providers did no better than those who self-assessed as moderate and poor in the use of interpreters [26]. Shriner and Hickey found that following completion of a training program for third year medical students in working with a translator there were significant changes in behavior. The most notable changes were: instructing the interpreter of their role, making eye contact with the patient for a majority of the encounter, using the first person and speaking directly to the patient, and avoiding side conversations with the interpreter [27]. Cha-Chi, et al. in a study of second year medical students found similar behavioral issues regarding working with an interpreter, including confidentiality and patient / translator positioning [28].

## Conclusions

This study’s major shortcoming is the small sample size. From the limited number of responses the findings cannot be extrapolated across all ACC member chiropractic college teaching clinics. Certain trends can be identified and highlight the need for further investigation. Further development of the survey instrument should include: inquiries into curriculum content specific to language and cultural discordance, the training of clinical supervisory staff in language and cultural issues and the content of specific written policies concerning non-English proficient patients.

The chiropractic profession continues to search for cultural authority and a larger influence in the health care system [29]. An integral part of that effort must be focused on reaching out to and providing services for the ever growing population for whom English is not the primary language spoken in the household. The chiropractic colleges should address that issue in their approaches to the non-English language proficient patient utilizing their teaching clinics, through a better understanding of best practices and a focus on cultural sensitivity.

The need to insure patient safety, effective and efficient treatment, appropriate and authentic informed consent and patient satisfaction require optimal patient - provider communication. The data confirm that while physician - patient cultural and language concordance is the optimal situation, this is often not practical. How to incorporate accurate and meaningful accommodations to address language disparity remains the challenge in all of health care, including the chiropractic profession.

## Competing interests

The author declare that they have no competing interests.

## Authors’ contributions

All authors read and approved the final manuscript.
